# Pan-Cancer Metastasis Prediction Based on Graph Deep Learning Method

**DOI:** 10.3389/fcell.2021.675978

**Published:** 2021-06-04

**Authors:** Yining Xu, Xinran Cui, Yadong Wang

**Affiliations:** Department of Computer Science, Harbin Institute of Technology, Harbin, China

**Keywords:** pan-cancer analysis, cancer metastasis, machine learning method, GCN, CNN

## Abstract

Tumor metastasis is the major cause of mortality from cancer. From this perspective, detecting cancer gene expression and transcriptome changes is important for exploring tumor metastasis molecular mechanisms and cellular events. Precisely estimating a patient’s cancer state and prognosis is the key challenge to develop a patient’s therapeutic schedule. In the recent years, a variety of machine learning techniques widely contributed to analyzing real-world gene expression data and predicting tumor outcomes. In this area, data mining and machine learning techniques have widely contributed to gene expression data analysis by supplying computational models to support decision-making on real-world data. Nevertheless, limitation of real-world data extremely restricted model predictive performance, and the complexity of data makes it difficult to extract vital features. Besides these, the efficacy of standard machine learning pipelines is far from being satisfactory despite the fact that diverse feature selection strategy had been applied. To address these problems, we developed directed relation-graph convolutional network to provide an advanced feature extraction strategy. We first constructed gene regulation network and extracted gene expression features based on relational graph convolutional network method. The high-dimensional features of each sample were regarded as an image pixel, and convolutional neural network was implemented to predict the risk of metastasis for each patient. Ten cross-validations on 1,779 cases from The Cancer Genome Atlas show that our model’s performance (area under the curve, AUC = 0.837; area under precision recall curve, AUPRC = 0.717) outstands that of an existing network-based method (AUC = 0.707, AUPRC = 0.555).

## Introduction

For years, tumor metastasis remains the leading cause of death from malignancies. Researchers have explored the causes of cancer metastasis based on a variety of biological processes (Spano et al., 2012; Fares et al., 2020; Wang et al., 2019). Gene expression data shows tumor status directly and is easily achieved. With its high sensitivity, wide detection range, and low cost, RNA-seq gene expression data is suitable for the analysis of biological samples from very limited sources like metastasizing cancer cells. For these reasons, RNA-seq gene expression data is widely used in cancer prognosis analysis and treatment plan formulation. Besides this, large-scale cancer databases like The Cancer Genome Atlas (TCGA), TCGA Pan-cancer Clinical Data Resource (TCGA-CDR) (Liu et al., 2018), and Cancer Cell Line E (Ghandi et al., 2019) are becoming more sophisticated, providing long-term-tracked reliable clinical data as well as the corresponding gene expression data and making it possible to conduct an in-depth study on the relationship between gene expression and clinical phenotype. Such genomic feature with cancer phenotype can effectively improve cancer prognosis outcome over the current clinical measures for risk assessment of patients (Byron et al., 2016; Grimes et al., 2018; Zhao et al., 2020a). The majority of cancer metastasis prediction methods based on RNA-seq gene expression data are feature processing followed by machine learning models.

### Cancer Metastasis Prediction Methods Based on Fold-Change Feature Selection

The commonly used fold-change feature selection methods include: *filters*, in which data is independent of the sequential machine learning model, and the method’s evaluation is to judge the relationship between the one-dimensional features and the target variables (i.e., Pearson correlation coefficient, Gini coefficient, information gain, variance check, and similarity measurement); *wrapper*s, in which feature selection is wrapped with the classifier, and the result of the classifier is used as the evaluation method of whether the feature is filtered or not (i.e., recursive feature elimination, stability selection); *embedded*, in which feature selection is carried out by the characteristics of the classifier (i.e., L1-regularization, L2-regularization, mean decrease impurity or mean decrease accuracy). Landemaine et al. (2008) selected six target genes according to gene expression fold-change and predicted breast cancer lung metastasis. Chibon et al. (2010) selected gene expression features according to *z*-scores and *P*-values and predicted sarcoma prognosis. GV Glinsky (2006) filtered gene expression features according to fold-change and predicted prostate cancer outcome. These methods ignored the biological significance in the data, leading to limited explanatory ability.

### Cancer Metastasis Prediction Methods Based on Priori Knowledge Feature Selection

Feature selection depends more on prior knowledge with the development of biological information database, including pathway database (i.e., KEGG, BioCyc, and Reactome) and gene enrichment database (i.e., MSigDB). Priori-knowledge-based cancer metastasis prediction methods mainly consist of two key steps: feature filtering based on priori-knowledge database or fold-change feature selection or both, then machine learning modeling (Kamps et al., 2017; Chaurasia et al., 2018; Ideta et al., 2021). These methods took gene pathway or enrichment knowledge into consideration but still ignored gene–gene regulation knowledge, from which vital information could be extracted. Besides this, it had been proven that regulation network information could enhance a machine learning model’s performance (Zhao et al., 2020b, 2021b). In conclusion, prior-knowledge-based feature extraction can promote a machine learning model’s performance albeit limitations still exist. Information from gene–gene regulation network should be effectively developed and efficiently combined with existing methods in future studies to achieve more convincing results.

### Cancer Metastasis Prediction Methods Based on Network Feature Extraction

Network information efficiently helps improve cancer outcome prediction and is always accompanied by prior knowledge feature selection. Protein–protein regulation information and gene regulation network could significantly improve a model’s prediction accuracy. In the study of Roy et al. (2014), features were first filtered by transcript factors’ prior knowledge, then ranked by protein–protein interaction network and gene regulation network, and finally predicted 13 cancer cases’ prognoses by support vector machine model. Protein interaction network is involved in the study of HY Chuang et al. (2007) and achieved 72.2% accuracy in prediction result on breast cancer metastasis by logistic regression classifier. In the study of Z Wang et al. (2020), application of a gene–gene regulation network-based feature extraction method helped raise the model’s area under the curve (AUC) from 0.623 to 0.707 on breast cancer outcome prediction. In conclusion, the network helped to increase the machine learning model’s performance. Meanwhile, the feature extracting method needs to be optimized.

### Machine Learning Models Used for Cancer Metastasis Prediction

Various machine learning models have been applied to cancer metastasis prediction. Tapak et al. (2015) predicted breast cancer survival and metastasis by multi-machine learning methods including naive Bayes, random forest, AdaBoost, support vector machine (SVM), least square SVM and Adabag, logistic regression, and linear discriminant analysis. They found that all these models performed well in cancer survival prediction but had a limited effect in cancer metastasis prediction. Nicolò et al. (2020) used random survival forest analysis to predict early-stage breast cancer metastatic relapse [area under receiver operating characteristic curve (AUROC) = 0.73]. Advanced machine learning is also applied to cancer metastasis prediction studies. Wang et al. (2020) classified a breast cancer patient’s metastasis status by support vector machine (AUROC = 0.71) and deep neural network (AUROC = 0.61). Among these studies, SVM outstands other models. The prediction results of advanced machine learning models like deep neural network (DNN) and convolutional neural network (CNN) were not satisfying. Until recently, DNN and CNN are used more commonly in combination with relational network prior knowledge. The research of Zhao et al.(2020c, 2021a) has proven the advanced machine learning models’ prediction performance.

### Application of Graph Convolutional Network in Network Feature Extraction

Graph convolutional network (GCN) is commonly used to handle topological data, including recovery of missing links or classification of ungrouped nodes. It is suitable for processing relation data including gene–gene regulation network, protein–protein interaction network, gene–disease relation network, *etc*. The research of Zhao et al. (2020c) indicated GCN’s prediction power on drug–target interaction networks. It is thus speculated that GCN is with high proficiency to handle gene–gene regulation network with improvement to directed network.

### Our Aims

To address the limitations of the above-mentioned approaches, we propose directed regulation graph convolutional network (DR-GCN), in which improvements for directed graph are added to GCN. In our study, gene–gene regulation network, gene–cancer correlation, and gene expression are integrated. Then, with the advanced feature we got from DR-GCN, we trained a CNN model to predict pan-cancer metastasis.

The major contributions of this research are as follows:

• We integrated multiple features by DR-GCN and reserved and extracted cancer-related information from gene expression data.
• The results of our CNN model show that DR-GCN feature extraction and CNN performed remarkably in pan-cancer outcome prediction.

## Materials and Methods

In this section, we first introduce the data preparation and workflow of the whole progress, then introduce the transfer formula of D-GCN-based feature extraction network (DR-GCN as well as its improvement on directed graph), and finally introduce the CNN model’s structure designed for pan-cancer outcome prediction.

### Data Preparation

In this study, we choose gene expression data (mRNA expression sequenced by second-generation technique) as our model’s input feature, which were downloaded from TCGA. To label the data, we analyzed clinical data downloaded from TCGA-CDR (Liu et al., 2018), which is the official clinical supplement for TCGA.

We choose three key clinical indicators from TCGA-CDR clinical file, which are tumor_status, new_tumor_event_type, new_tumor_event_site, and PFI.time; tumor_status tells whether one patient is in a state of with tumor, tumor-free, or not clear; new_tumor_event_type tells whether one patient’s new tumor event is metastasis, recurrence, new primary, or none; new_tumor_event_site tells whether one patient has a tumor discovered in a certain site; and PFI.time tells one patient’s last record time point or last tumor event time point, whichever is shorter. Firstly, three types of patients were labeled “metastasis” (1): cases that have a clear tumor_status metastasis, cases that have no clear tumor_status but have clear new_tumor_event_type metastasis, and cases that have no clear tumor_status or new_tumor_event_type label but have a clear new_tumor_event telling the metastasis site. Secondly, among the residual cases, we removed the cases in which the tumor_status is “not clear” or “NA” and then preliminarily label the remaining cases “non-metastasis” (0); among these “non-metastasis” cases, we kept those with new_tumor_event “NA” as well as tumor_status “tumor free.” Finally, we considered the PFI.time; some of the non-metastasis cases could be temporary due to the short clinical test interval. According to this consideration, we kept 70% of the longest non-metastasis cases and all the metastasis cases.

Considering the sample size, for a relatively balanced proportion of positive and negative cases, we choose six cancer types from TCGA for this study, which are as follows: breast invasive carcinoma, stomach adenocarcinoma, lung squamous cell carcinoma, lung adenocarcinoma, pancreatic adenocarcinoma, and skin cutaneous melanoma as pan-cancer input. These six cancer types have the highest rate of clearly described metastasis status in TCGA-CDR. Besides that, they have a moderate proportion of positive and negative cases (near 1:1). They are also among the most common human cancers. The number of positive and negative cases for each cancer is shown in Table 1.

**TABLE 1:** Number of positive and negative cases for each cancer type.

	Non-metastasis	Metastasis
	cases (0)	cases (1)
Breast invasive carcinoma	589	102
Lung adenocarcinoma	152	122
Lung squamous cell carcinoma	157	76
Stomach adenocarcinoma	151	99
Skin cutaneous melanoma	75	153
Pancreatic adenocarcinoma	21	82
Total	1,145	634

**FIGURE 1:**
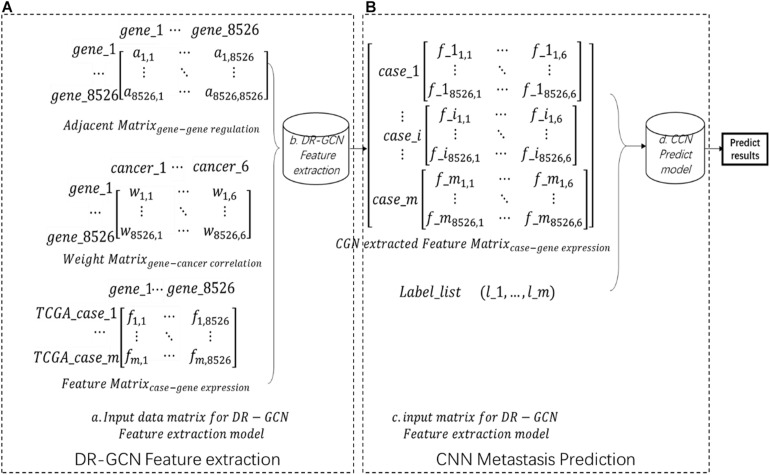
Workflow **(A)** showing the input data matrix for directed relation-graph convolutional network (DR-GCN). Then, data were put into a DR-GCN feature extraction model. After feature extraction, we get a graph–structure data matrix as shown in panel **(B)**. Then, this data matrix combined label were put into a convolutional neural network prediction model. Finally, we got the prediction result.

Each case’s gene expression feature is listed in a feature matrix, in which *f*_ij_ presents *gene*_j_’s expression quantity in *case*_i_ (Figure 1A). The gene regulation network here is downloaded from humannet V2 (Ideta et al., 2021); an adjacency matrix of the gene regulatory network was obtained, A total of 17,926 genes were included in this network, in which *a*_ij_ = 1 presents *gene*_i_ regulates *gene*_j_ (Figure 1A). The initial feature of each gene, which is gene’s correlation with certain cancer types, is downloaded from DisGenet (Glinsky, 2006), and then we made a gene–cancer correlation table in which *w*_ik_ = 1 presents *gene*_i_ and *cancer*_k_ is related (Figure 1A. Then, we kept the intersection genes and filtered the data (Figure 1A).

### Workflow

After the data preparation process, we extracted advanced feature from pan-cancer gene expression data by relational graph convolutional network (R-GCN)-based model DR-GCN with improved model’s ability for directed graph. In this step, data’s dimension is increased, the whole dataset is shaped (m,1,8526,6), in which m presents m cases included in our study, 1 presents one-layer graph structure, and 8526,6 presents the two-dimension feature extracted by DR-GCN (Figure 1B). Finally, based on the feature and label prepared, we designed and trained a CNN model and made prediction of pan-cancer outcomes. The whole workflow is shown in Figure 1.

### R-GCN-Based Gene Expression Feature Extraction

R-GCN was introduced and applied to network link prediction (recovery of missing facts) and entity classification (recovery of missing entity attributes) (Kipf and Welling, 2016; Schlichtkrull et al., 2018). In this study, R-GCN is applied to handle gene–gene regulation network. More than ever, gene–gene regulation network is a directed network, and R-GCN was generally applied to undirected graph. To address this issue, we apply DR-GCN, a novel directed-graph processing method that has been added in this study.

#### DR-GCN Model Architecture

For gene–gene regulation network G = (V,E), we use binary adjacency matrix A, as described in data preparation. The adjacency matrix shows the associative property of the graph; to add the nodes’ self-connection feature, we add identity matrix to the adjacency matrix:

(1) $$\tilde{A}=A+I$$

A graph convolution layer can be written as such a nonlinear function:

(2) $$H^{l+1}=f\,\left(H^{l},\tilde{A}\right)$$

in which *H*_0_ = *A*. Graph convolution problems can be abstracted as solution of *f*(*H*^*l*^,*A*) = σ(*AHW*^(*l*)^), in which *W*^(*l*)^is the graph feature, *H* is weight, and σ is an activation function, $H_{0}\;=\tilde{A}$.

Here we consider our model with the following layer-wise propagation rule:

(3) $$H^{\left(l+1\right)}=\sigma \,\left({\tilde{D}}^{-1}\,\tilde{A}\,H^{\left(l\right)}\,W^{\left(l\right)}\right)$$

We consider that all genes have equal weight; then, we have:

(4) $$H^{\left(l+1\right)}=\sigma \,\left({\tilde{D}}^{-1}\,\tilde{A}\,H^{\left(l\right)}\right)$$

*H*^(*l*)^ denotes the feature vector of each gene as described in “Data Preparation.” $\tilde{D}$ is the diagonal node degree matrix of $\tilde{A}$. We have $\tilde{D}=\sum_{j}{\tilde{A}}_{\mathrm{ij}}$. $L^{R\,W}={\tilde{D}}^{-1}\,\tilde{A}=I_{n}-D^{-1}\,A$ is Laplacian.

After the DR-GCN feature extraction, a new feature matrix contains all gene–cancer information as well as gene–gene regulation network information. In the following section, we show that the form of this propagation rule can be applied on directed graphs.

#### Directed-Graph Processing Method

For the directed graph, we use each nodes’ out-degree to initialize the degree matrix *D*. In order to make this matrix full rank, we have a strategy as follows:

$$D_{\text{ji}}=\{\begin{array}{cc} \begin{array}{c} \frac{1}{\sum D\,i\,n}\\ -\frac{1}{\sum D}\\ 0 \end{array}\; & \begin{array}{c} i\,f\,D_{\text{ij}}\neq 0\,\text{and}\,D\,i\,n_{\text{ij}}\neq 0\\ i\,f\,D_{\text{ij}}\neq 0\,\text{and}\,D\,i\,n_{\text{ij}}=0\\ \text{otherwise} \end{array} \end{array}$$

$\frac{1}{\sum D\,i\,n}$ is the normalization operator for directed graph. We use in-degree in this study; $-\frac{1}{\sum D}$ is minimal value added to the degree matrix. In this way, we keep the directivity of the graph and also guarantee the invertibility of the Laplace matrix.

### CNN-Based Classification Model

After the feature extraction by DR-GCN, a CNN model is designed and used as a supervised machine learning model to classify TCGA pan-cancer as benign or malignant.

**FIGURE 2:**
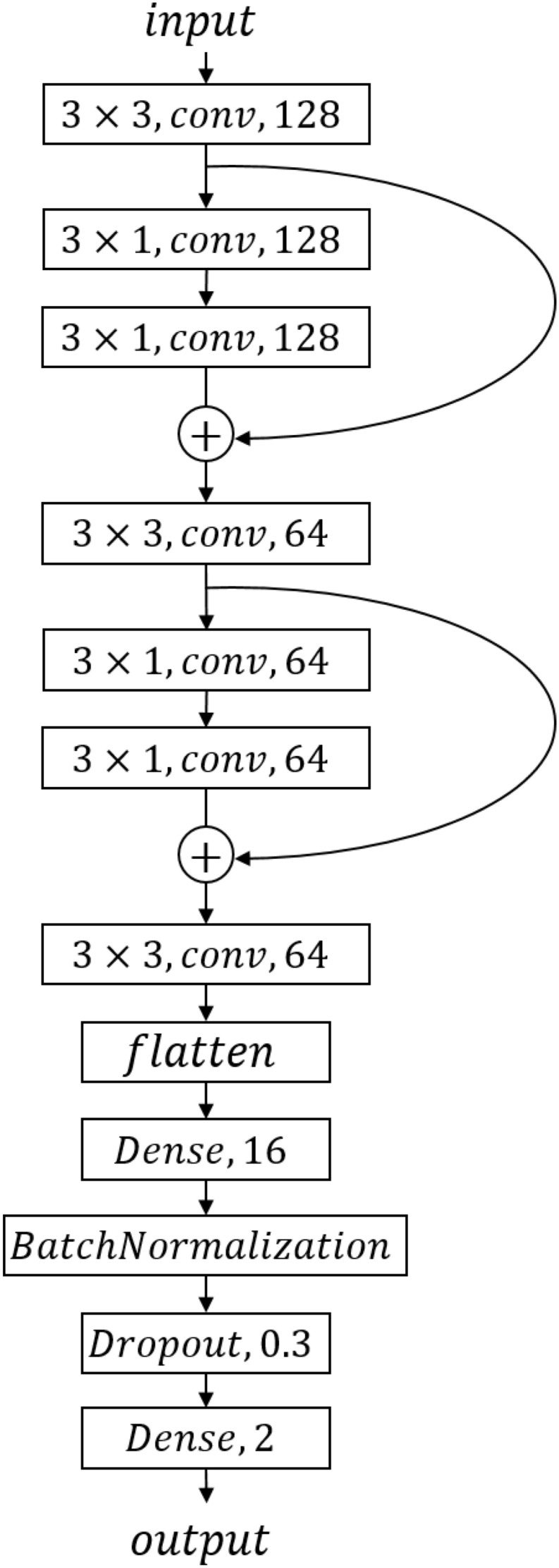
Convolutional neural network structure and parameters.

We designed a CNN model, the structure of which is shown in Figure 2. We got one input layer, seven convolution layers followed by activation layers, one flattened layer, one batch-normalized layer, and two dense layers with one drop layer in CNN. LeakyReLU is chosen as each convolution layer’s activation function to avoid neuronal death; the parameter of LeakyReLU is 0.1. We converted the two-category result to one-hot format and used softmax activation function for the last dense layer to achieve a two-category prediction result. Each layer’s parameters are shown in Figure 2.

We choose categorical_crossentropy as loss function and optimizer “adam.” This pair worked better in our study than the more commonly used binary_cross_entropy and “RMSProp” optimizer in our two-category data.

## Experiments

In this section, we first generated a real-world gene–gene regulation network, set the initial weight feature of each gene as the gene–cancer correlation, then put them into DR-GCN, and run the feature extraction module. After DR-GCN, we got a gene–gene regulation that masked the gene–cancer correlation weight table, and then we weighed the pan-cancer gene expression data from TCGA by the gene–cancer correlation weight table. Finally, we designed and trained a CNN model to identify the malignancy of the tumor.

### Experiments on Feature Extraction

The gene–gene regulation network used here was downloaded from humannetV2 (Hwang et al., 2019), and the initial weight feature of each gene is downloaded from DisGenet (Piñero et al., 2020). With a raw network gene list, we converted all gene names to gene symbol ID, and then an intersection was made between raw TCGA FPKM gene list and raw network gene list. Finally, 8,526 overlapping unique genes derived from the two gene lists were included in this study.

The patients were classified as non-metastasis and metastasis groups based on the clinical information from the TCGA-CDR. After data processing, we got 1,145 non-metastasis cases and 634 metastasis cases from six different cancer types (shown in Table 1). All the cases were mixed randomly, and we generated our final dataset.

We next input gene–gene interaction network (8526,8526) and gene–cancer (8526,6) initial weight into DR-GCN. After DR-GCN feature extraction, we got the gene–gene interaction network that masked the gene–cancer relation weight (8526,6). Then, we multiplied pan-cancer’s gene expression data by this weight as CNN’s input in the following experiment.

### Experiments Across Pan-Cancer Datasets

We applied the CNN model to predict the cancer outcome on pan-cancer data. In the training progress, we choose categorical_crossentropy as loss function and optimizer “adam.” This pair worked better in our study than the more commonly used binary_cross_entropy and “RMSProp” optimizer in our two-category data. In modeling progress, we adjusted the model’s parameter by AUROC as performance measure. After parameter adjustment and model structure adjustment, we got a CNN model structure as well as each layer’s parameters as shown in Figure 2.

To evaluate the prediction power of the workflow and the models described above, we performed a 10-fold cross-validation (CV) on pan-cancer data with ninefold data for training and onefold data for test. The classification performance was evaluated on the test data in each dataset separately.

**FIGURE 3:**
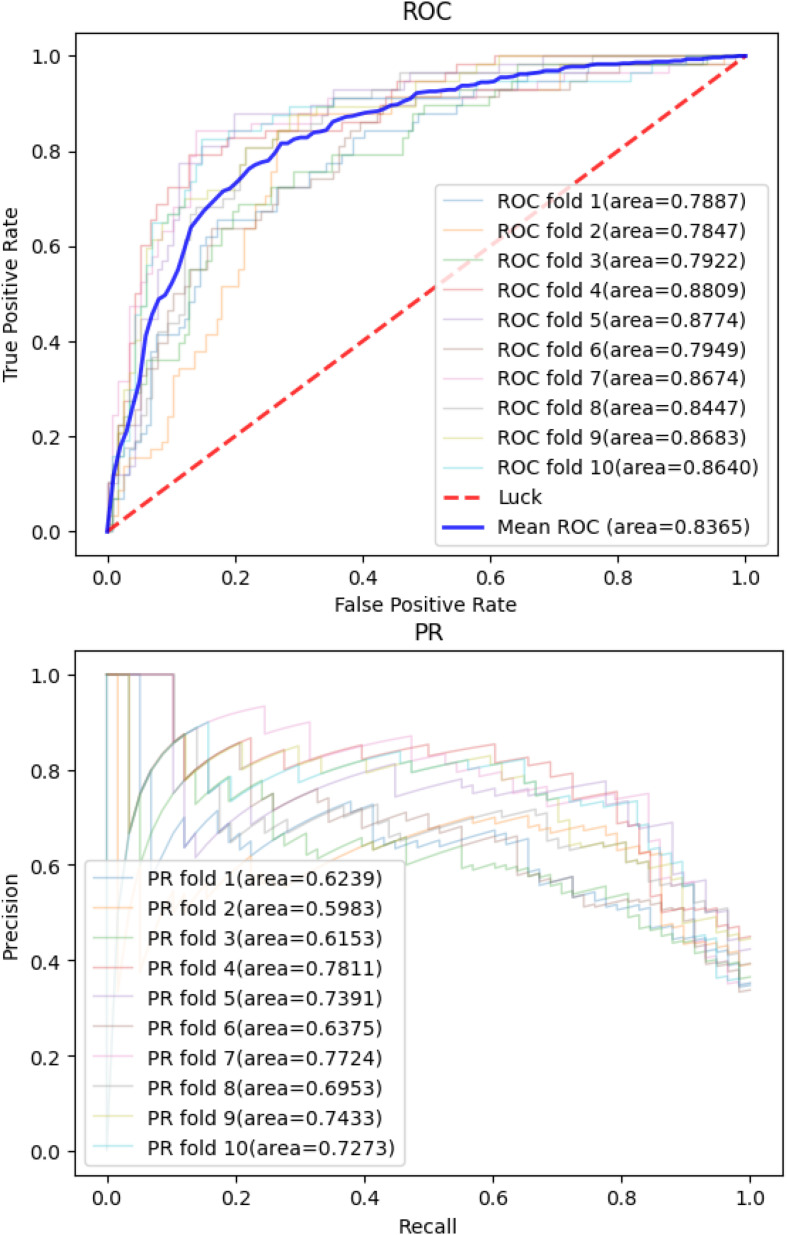
Convolutional neural network model’s receiver operating characteristic curve and precision recall curve in 10-fold cross-validations.

Then, we applied the same pan-cancer data and experimental process on the network-based model of Wang et al. (2020) to demonstrate our model’s predictive ability compared with those of other network-based models. We report 10CVs’ mean AUROC and the area under precision recall curve (AUPRC) scores of each model mentioned above in Table 2. Our CNN model’s 10-CV receiver operating characteristic curve and precision recall results are shown in Figure 3.

**TABLE 2:** Comparison of prediction models on pan-cancer data.

	AUROC	AUPRC
DR-GCN-CNN	0.8365	0.7164
NetML-SVM	0.6122	0.4837
NetSML	0.6396	0.6331

## Discussion and Conclusion

For years, researchers made efforts to enhance the cancer metastasis predicting models’ performance. Many methods have been raised for different cancers’ metastasis prediction, but the prediction results of the models were not satisfying, especially in pan-cancer metastasis studies; few of them took gene–gene regulation network into consideration. We believe that gene–gene regulation network information provides vital information in a pan-cancer metastasis study. According to this consideration, we applied DR-GCN to extract feature from gene–gene regulation net and achieved high AUC and AUPR scores in a CNN predicting model.

In most studies, researchers used prior knowledge like gene co-expression information and pathway analysis in feature selection to enhance the cancer metastasis predicting models’ performance. In contrast, our work applied an effective R-GCN method, DR-GCN, specially for gene–gene regulation network information extraction. We efficiently added information to regular gene expression data and thereby achieved a better CNN prediction result, but limitations still exist in DR-GCN feature extraction and similar researches. DR-GCN essentially highlighted nodes that could be vital according to information network; its reliability highly depends on the network’s reliability. Due to DR-GCN’s character mentioned above, as well as the gene–gene regulation network which remained to be improved, we believe that DR-GCN’s performance is far from optimal. In subsequent studies, R-GCN can be applied to gene–gene regulation focusing on unknown regulation relation detection.

In cancer metastasis predictions, the most commonly used predicting model is SVM, and it always worked better than advanced models like DNN and CNN because SVM is more suitable for linear gene expression data. DR-GCN feature extraction provided a higher feature dimension and therefore enhanced CNN model’s prediction performance.

In conclusion, DR-GCN feature extraction distinctly improved the CNN model’s prediction ability compared with other cancer metastasis prediction methods. Ten-fold cross-validation confirmed the high AUC and AUPR of CNN. The code and results of DR-GCN and CNN model are uploaded on Github^1^, which will allow researchers to apply it to other pan-cancer datasets.

## Data Availability Statement

The datasets presented in this study can be found in online repositories. The names of the repository/repositories and accession number(s) can be found in the article/supplementary material.

## Author Contributions

YX designed workflow, improved feature extraction algorithm and structured predict model, then processed main part of experiment, and composed main part of this thesis. XC participated predict model’s parameter debugging and thesis composing. YW advised the whole thesis work. All authors contributed to the article and approved the submitted version.

## Conflict of Interest

The authors declare that the research was conducted in the absence of any commercial or financial relationships that could be construed as a potential conflict of interest.

## Footnotes

1 https://github.com/YININGXU512/GCN-CNN
